# Urinary microbiome in renal transplant patients with BK polyomavirus reactivation

**DOI:** 10.1099/jmm.0.002196

**Published:** 2026-09-07

**Authors:** Kieran A. Bates, Viviam B. Rivera, Daniel Glicklich, Thomas Diflo, Vishnu Chaturvedi, Rajat Nog

**Affiliations:** 1 Blizard Institute, Faculty of Medicine and Dentistry, Queen Mary University of London, London, UK; 2 Department of Medicine, Westchester Medical Center, Valhalla, NY, USA; 3 Department of Surgery, Westchester Medical Center, Valhalla, NY, USA; 4 Department of Pathology, Microbiology and Immunology, New York Medical College, Valhalla, NY, USA; 5 Department of Pathology and Laboratory Medicine, Westchester Medical Center, Valhalla, NY, USA

**Keywords:** BK polyomavirus, microbiome, transplantation

## Abstract

**Introduction.** BK polyomavirus (BKPyV) reactivation is a significant health risk among renal transplant recipients that can lead to nephropathy and allograft loss.

**Hypothesis/Gap statement.** While the microbiota is increasingly recognized as an important determinant of viral infection and pathogenesis, as well as itself undergoing compositional changes in response to infection, the urinary microbiome has yet to be investigated in the context of BK polyomavirus reactivation.

**Aim.** This study aimed to investigate associations between the urinary microbiome and BKPyV-DNAemia in renal transplant patients.

**Methodology.** Shotgun metagenomics of the urinary microbiome was conducted for 22 renal transplant recipients, 11 of whom had BKPyV-DNAemia. Sequence data were analysed using two complementary approaches to identify common microbiome associations with BKPyV-DNAemia: (1) Kaiju – a DNA-to-Protein method that captures bacteria, archaea, fungi, microeukaryotes and DNA viruses and (2) MetaPhlAn4 – a DNA-to-Marker method using a reference database of specific marker genes of prokaryotes.

**Results.** We found increased observed diversity of bacterial taxa in control patients compared to those with BKPyV-DNAemia for data analysed with MetaPhlAn4 (*P*=0.037) but not Kaiju (*P*>0.05), which followed a similar trend. Significant differences in microbial beta diversity between the control and BKPyV-DNAemia patient group were identified for the Kaiju dataset (*P*=0.027) but not for MetaPhlAn4 (*P*>0.05), with viral reads likely driving these differences in the Kaiju dataset. Both Kaiju and MetaPhlAn4 identified *Proteobacteria*, *Firmicutes* and *Actinobacteria* as bacterial phyla with greatest relative abundance across samples. Screening bacterial species data generated from Kaiju and MetaPhlAn4 against a database of 243 human pathogens identified 8 pathogenic species recovered from both datasets that were present in the urinary microbiome of renal transplant patients.

**Conclusion.** The observed evidence for differences in microbiome diversity and composition associated with BKPyV-DNAemia may play an important role in its pathology and guide the development of diagnostic biomarkers. Our findings warrant further investigation across larger patient cohorts that are more evenly balanced for gender.

## Data summary

 Sequence data are uploaded under BioProject ID PRJNA1252894. Sample accessions are listed in Table S2.

## Introduction

BK polyomavirus (BKPyV) reactivation defined as BKPyV-DNAemia >10,000 c ml^−1^ is a major driver of post-renal transplant nephropathy [1, 2]. Usually contracted in childhood, BKPyV is carried by at least 80% of adults, where it lies dormant in the renal–urinary tract [3, 4] with little clinical impact in healthy individuals [5, 6]. Among immunosuppressed patients; however, BKPyV may reactivate, often with severe pathological consequences [5, 7]. Kidney transplant recipients are commonly affected by BKPyV reactivation, with a median of 19.5% (range: 8–62%) of patients across published studies experiencing post-transplantation viremia [6]; some patients subsequently develop polyomavirus-associated nephropathy, ureteral stenosis and a heightened risk of allograft loss [6, 8–10]. Screening for BKPyV reactivation in kidney transplant recipients by quantitative PCR of urine/peripheral blood samples has consequently become a mainstay in clinical management [11, 12].

Though most BKPyV reactivation post-transplantation is in the form of low-grade viruria and is usually controlled by the host immune system, the risk factors responsible for uncontrolled reactivation are largely unknown or have weak scientific evidence [13]. Once considered sterile, advances in DNA sequencing and culture methods have revealed that urine harbours a microbiota that may play important functions in human health [14–16]. Yet, despite recognition of the role of microbes in enhancing or suppressing viral pathogens [17–21], the potential importance of urinary microbial communities in BKPyV reactivation has not been explored. One prior study showed associations between the gut microbiome and BKPyV infection in renal transplant recipients, with a significant increase in the Firmicutes/Bacteroidetes ratio in BKPyV reactivated patients compared to controls [22]. However, microbial interactions at the site of viral shedding (the urinary epithelium) that are likely to be key in shaping disease are not known. Mounting evidence suggests that the urinary microbiota is important in various renal transplant-associated pathologies, including interstitial fibrosis and tubular atrophy [23] and allograft dysfunction [24]. The urinary microbiome has also been shown to modulate immunity and inflammation [25, 26], which may be key in the progression of BKPyV reactivation. Exploring the urinary microbiome may, therefore, not only progress our understanding of BKPyV reactivation but also potentially yield novel microbial biomarkers linked to disease progression and pathology.

We conducted a study involving 22 renal transplant recipients, of which 50% had BKPyV reactivation, with the control group having undergone transplantation without concurrent BKPyV reactivation. Using shotgun metagenomics, we assessed the urinary profiles of this patient cohort to investigate possible links between the microbiome and BKPyV reactivation. We found evidence for both higher bacterial taxonomic alpha diversity in control versus BKPyV reactivated patients and differences in taxonomic beta diversity between patient groups. Finally, using a database of 243 bacterial pathogens, we identified clinically relevant taxa present in the urinary microbiome of this renal transplant patient cohort.

## Methods

### Sample collection

The study protocol was approved by the Institutional Review Board of Westchester Medical Center. After obtaining informed consent, a cohort of 22 patients was recruited into the study. Of the 22 subjects, 11 were patients with significant BKPyV reactivation with BKPyV-DNAemia >10,000 c ml^−1^ [presumptive BK Polyomavirus-associated nephropathy (BKPyVAN)] and the remaining 11 we classified as controls (undetected BKPyV-DNAemia) matched to age, gender and time since transplant. We defined the BKPyVAN reactivation group as patients who had >4 log_10_ c ml^−1^ BKPyV‐DNAemia and triggered immunosuppression reduction for presumptive BKPyVAN. Patient charts were reviewed to acquire clinical and demographic information relevant to the study. Data collected included demographics, such as age at the time of transplant and sex. Clinical data included the cause of end-stage renal disease (ESRD), requirement and duration of haemodialysis (HD) pre-transplant, type of transplant (organs collected, living or deceased donor), induction and maintenance immunosuppression regimen, the requirement for a stent at transplantation, delayed graft function (DGF) post-transplant, rejection episodes and treatment post-transplant, change in renal function with rejection, episodes of neutropenia or lymphopenia post-transplant, episodes of CMV viremia post-transplant, timing and duration of BKPyVAN and its effect on renal function post-transplant. Blood and urine samples were collected simultaneously from all patients for the detection of BKPyV-DNAemia by Quantitative Real-Time PCR using a Roche Cobas 6800 BKV System (Roche, Basel, Switzerland). All samples were stored at −80 °C for analysis. Routine kidney biopsy was performed in patients with clinical indications (suspicion of rejection or BKPyVAN).

### Clinical characteristics of patients

Of the 22 patients enrolled, 20 patients (90.9%) were male and 2 (9.1%) were female. The mean age of patients was 54.3 years at the time of transplantation. The cause of renal failure requiring transplantation was diabetes and HTN in 27.2%, HTN in 13.6% and cardio-renal syndrome (CRS) in 9.1% of patients, with other causes in the remaining patients (Table 1). Eighteen (81.8%) of the patients were on HD prior to transplantation with a mean period of 4.6 years before transplant. Eighteen (81.8%) patients had a kidney transplant alone, three (13.6%) patients had a dual heart-kidney transplant and one (4.5%) patient had a dual liver-kidney transplant. Six (27.3%) of the patients had a living related donor and sixteen (72.7%) were from deceased donors. Post-transplant DGF was observed in three patients (27.3%) among the case group compared to six (54.5%) in the control group. The mean time since transplantation and development of BKPyV-DNAemia was 7.6 months. Five patients had biopsy-proven BKPyV nephropathy. Eleven cases (100.0%) and ten controls (90.9%) received thymoglobulin induction immunosuppression and one patient (4.5%) received basiliximab induction. Post-transplant, standard maintenance immunosuppression used for both cases and controls was tacrolimus, mycophenolate and prednisone. However, at the time of sample collection, seven cases (63.6%) and eight controls (72.7%) were on tacrolimus and mycophenolate, one case (9.1%) and one control (9.1%) were on tacrolimus, mycophenolate and prednisone, one case (9.1%) and two controls (18.2%) on cyclosporine alone, one case (9.1%) on belatacept alone and one case (9.1%) was on mycophenolate, prednisone. Five cases (45.5%) and two controls (18.2%) had at least one rejection episode post-transplant. The most common intervention for BKPyV-DNAemia was immunosuppression reduction, starting with mycophenolate reduction. At the time of sample collection, one patient (9.1%) indicated urinary tract infection (UTI) symptoms in the control group and tested positive for *Escherichia coli* from urine culture. Four cases (36.4%) and six controls (54.5%) had at least one episode of neutropenia post-transplant and ten cases (90.9%) and eight controls (72.7%) had lymphopenia post-transplant.

**Table 1.** Demographics of study populations

Characteristic	Control patient (*n*=11)	BKPyV-DNAemia patient (*n*=11)
Age in years (range)	54 (27–74)	54.5 (32–72)
Male sex, *n* (%)	10 (90.9%)	10 (90.9%)
Kidney transplant *n* (%)	10 (90.9%)	8 (72.7%)
Liver–kidney transplant *n* (%)	1 (9.1%)	0 (0%)
Heart–kidney transplant *n* (%)	0 (0%)	3 (27.3%)
Living-related donor *n* (%)	2 (18.2%)	4 (36.4%)
Deceased donor *n* (%)	9 (81.8%)	7 (63.6%)
ESRD cause: DM and HTN *n* (%)	4 (36.4%)	2 (18.2%)
ESRD cause: Unknown *n* (%)	2 (18.2%)	1 (9.1%)
ESRD cause: Lithium *n* (%)	1 (9.1%)	0 (0%)
ESRD cause: EtOH/ESLD/HRS *n* (%)	1 (9.1%)	0 (0%)
ESRD cause: DM and obesity *n* (%)	1 (9.1%)	0 (0%)
ESRD cause: HIV *n* (%)	1 (9.1%)	0 (0%)
ESRD cause: HTN *n* (%)	1 (9.1%)	2 (18.2%)
ESRD cause: CRS *n* (%)	0 (0%)	2 (18.2%)
ESRD cause: SLE *n* (%)	0 (0%)	1 (9.1%)
ESRD cause: IgA Nephropathy *n* (%)	0 (0%)	1 (9.1%)
ESRD cause: FSGS *n* (%)	0 (0%)	1 (9.1%)
ESRD cause: AKI post-heart transplant *n* (%)	0 (0%)	1 (9.1%)
Requirement of HD *n* (%)	9 (81.8%)	9 (81.8%)
Duration of HD	4.5 years	2.7 years
Timing of BK Viremia from transplantation	na	7.8 months
Duration of BK Viremia	na	20.1 months

AKI, acute kidney injury; DM, diabetes mellitus; ESLD, end-stage live disease; FSGS, focal segmental glomerulonephritis; HRS, hepato-renal syndrome; HTN, hypertension; NA, not applicable; SLE, systemic lupus nephritis.

### DNA extraction and shotgun metagenomic sequencing

DNA extraction, library preparation and sequencing were conducted by GENEWIZ, LLC. (South Plainfield, NJ, USA). DNA was extracted from urine samples using the QIAmp Circulating Nucleic Acid Kit (Qiagen, Germantown, MD, USA) following the manufacturer’s instructions. Extracted DNA was quantified using a Qubit 2.0 Fluorometer (Life Technologies, Carlsbad, CA, USA) and the quality was checked with TapeStation 4200 (Agilent Technologies, Palo Alto, CA, USA). A NEB NextUltra DNA Library Preparation kit was used following the manufacturer’s recommendations (Illumina, San Diego, CA, USA). Briefly, genomic DNA was fragmented by acoustic shearing with a Covaris S220 instrument and subsequently end-repaired and adenylated. Adapters were ligated after adenylation of the 3′ ends. Adapter-ligated DNA was indexed and enriched by limited cycle PCR. The DNA library was validated using TapeStation 4200 (Agilent Technologies, Palo Alto, CA, USA) and was quantified using Qubit 2.0 Fluorometer (Life Technologies, Carlsbad, CA, USA). Sequencing was conducted using a HiSeq instrument (Illumina, San Diego, CA, USA) following a 2×150 bp paired-end strategy.

### Bioinformatics and statistical analysis

Samples averaged 74.6 million paired reads per sample (SD 21.8 million). Quality control, including adaptor trimming, filtering of low-quality reads and removal of human reads was conducted using KneadData [27] (https://huttenhower.sph.harvard.edu/kneaddata/) with default settings. Since determining taxonomic classification and relative abundance can vary greatly depending on the choice of metagenomic tool and database used [28–30], we employed two different taxonomic classifiers, enabling comparisons to be made. Kaiju [31] is a DNA-to-Protein method which compares metagenomic sequence reads with genomic databases of protein-coding sequences. Conversely, MetaPhlAn4 [32] is a DNA-to-Marker method which uses a reference database of specific marker genes. For MetaPhlAn4 [32], taxonomic composition was determined using the mpa_vJan21_CHOCOPhlAnSGB_202103 database. Bacteria, archaea, microeukaryotes, fungi and DNA viruses were identified with Kaiju [31] using the NCBI blast nr_euk 2023-05-10 database in greedy mode. Data obtained from MetaPhlAn4 and Kaiju were imported into R (v4.5.2) and analysed using the phyloseq package (v1.54.0) [33]. Data were filtered to include taxa with a mean relative abundance >0.1%. Reads unclassified to Kingdom level were removed prior to analysis. Observed taxa richness was calculated and compared between BKPyV-DNAemia and control groups using non-parametric Wilcoxon rank-sum tests. For Kaiju analysis, reads were also rarefied to the lowest read depth across samples to confirm that the results were robust to differences in sequencing depth. Taxonomy data generated from Kaiju and MetaPhlAn4 were centred log-ratio transformed and beta diversity was visualized using principal components analysis using the packages microbiome (v1.32.0) [34] and MixOmics (v6.34.0) [35]. Statistical differences in beta diversity were tested using permutational multivariate analysis of variance (PERMANOVA) with 10,000 permutations, using the adonis2 function from the Vegan package (v2.7.2) [36]. For Kaiju, analyses of the filtered dataset were performed for all microbes (i.e. including viral reads) as well as for bacterial reads only (viral reads excluded). Differential abundance analysis for microbial taxa was conducted using ALDEx2 (v1.42.0) [37]. Potential bacterial pathogens within the filtered MetaPhlAn4 and Kaiju datasets were identified by screening against a database of known human bacterial pathogens compiled from CZID [38]. Pathogens identified by both methods were then reported.

## Results

**Fig. 1.**
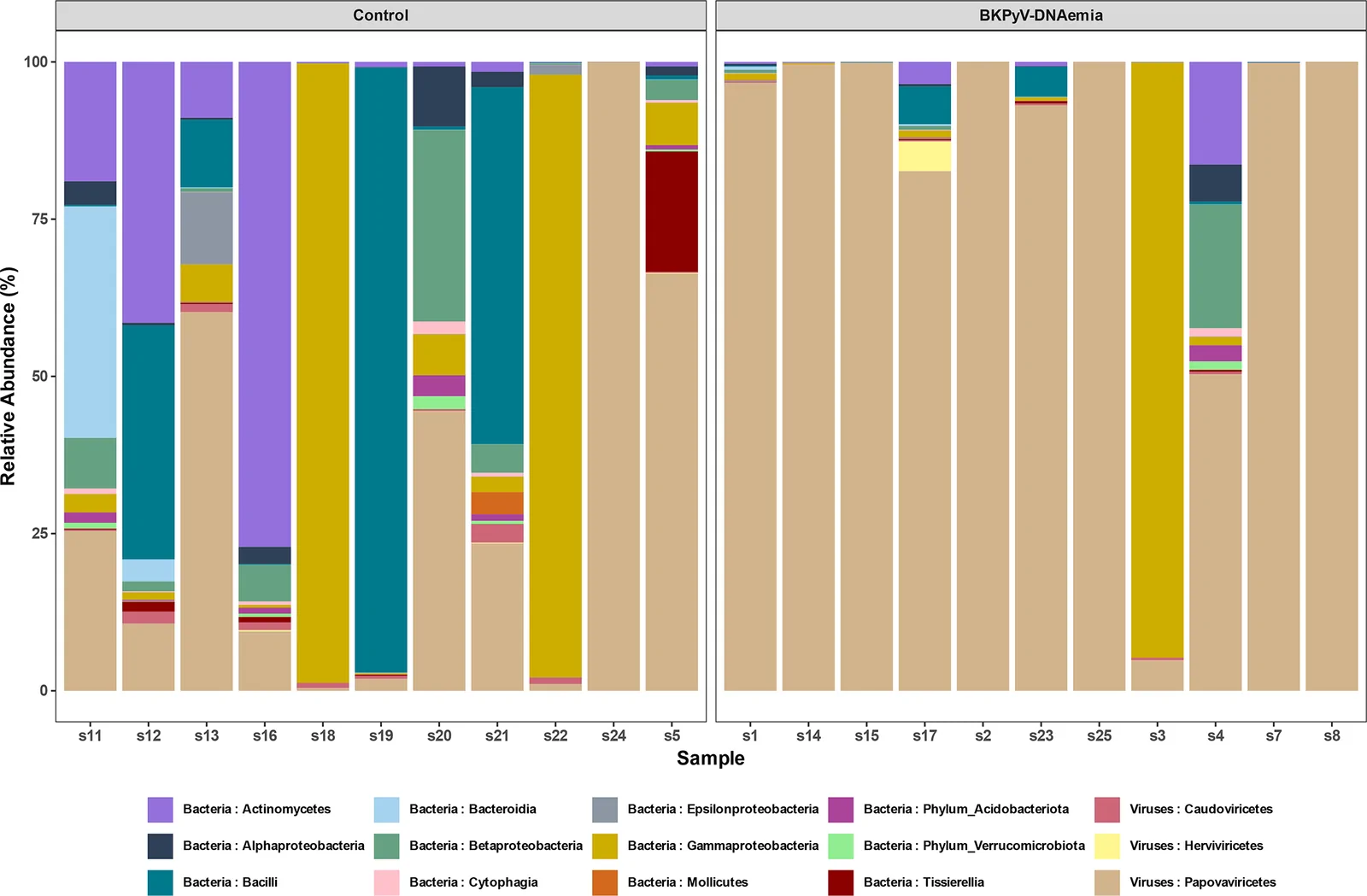
Stacked bar plot demonstrating the relative abundance of microbial taxa (mean relative abundance >0.1%) at the class level from Kaiju analysis. *N*=22 (11 control, 11 BKPyV-DNAemia).

**Fig. 2.**
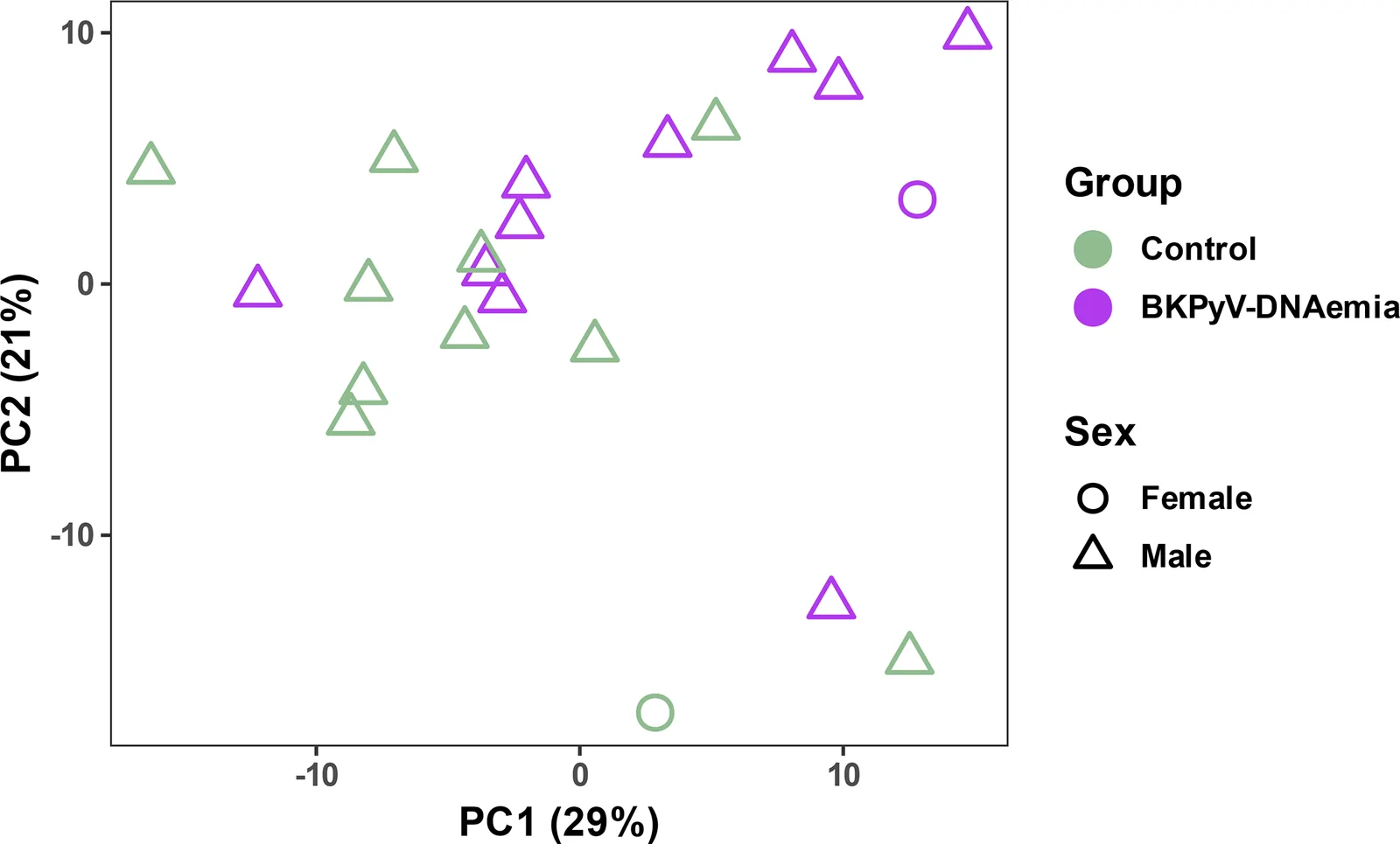
PCA of CLR transformed data from Kaiju output in control and BKPyV-DNAemia patients. *N*=22 (11 control, 11 BKPyV-DNAemia).

Taxonomic profiling using Kaiju yielded 29,063 classified microbial taxa, of which 51 species had a mean relative abundance greater than 0.1% across control and BKPyV-DNAemia patients. Most classified reads were assigned as viral, with *Betapolyomavirus* the most abundant genus. Dominant bacterial phyla included the *Firmicutes*, *Proteobacteria* and *Actinobacteria* (Figs 1 and S1, available in the online supplementary material). MetaPhlAn4 identified a total of 114 bacterial species, of which 43 taxa had a mean relative abundance >0.1%. Phylum-level classification supported results from Kaiju, with members of the *Proteobacteria*, *Firmicutes* and *Actinobacteria* having the greatest relative abundance (Fig. S2). For Kaiju, 29 taxa were present in all samples, with all 51 taxa found in 45% of samples (Fig. S3). Few bacterial taxa identified by MetaPhlAn4 were shared across samples, with only a single species (*Serratia marcescens*) recorded in 86% of patients (Fig. S3). For MetaPhlAn4 taxonomic profiles, observed bacterial species diversity was significantly higher in control patients than the BKPyV-DNAemia group (Wilcoxon rank-sum test W=92.5, *P*=0.037) but not for Kaiju, even with non-bacterial taxa removed (Wilcoxon rank-sum test bacteria only: W=86, *P*=0.097, all microbes: W=87.5, *P*=0.078, Fig. S4), although a trend of increased alpha diversity in the controls was observed. Taxonomic beta diversity significantly differed based on BKPyV reactivation status for the Kaiju dataset (PERMANOVA F_(1,20)_ = 2.23, R^2^=0.10, *P*=0.027, Fig. 2) but not for MetaPhlAn4 data (*P*>0.05). Analysis of only bacterial reads from the Kaiju data found no significant difference in taxonomic beta diversity (PERMANOVA *P*>0.05) indicating that observed differences in the full dataset were driven by viral taxa. ALDEx2 analysis of the Kaiju dataset identified four taxa with significantly higher relative abundance in the BKPyV-DNAemia patients (*Betapolyomavirus hominis*, unclassified *Polyomaviridae*, unclassified *Betapolyomavirus* and *Betapolyomavirus secuhominis* (JC virus), Table S1). No significant differentially abundant taxa were identified by ALDEx2 for the MetaPhlAn4 dataset. Screening MetaPhlAn4 and Kaiju data subset to bacteria against a list of known human pathogens identified a total of eight bacterial taxa detected by both methods (Table 2). Of these eight taxa, a greater number of pathogens were detected in control patients with MetaPhlAn4 but did not differ for Kaiju.

**Table 2.** Prevalence of human bacterial pathogens identified in both MetaPhlAn4 and Kaiju datasets

Pathogen	Prevalence (%) control patient (*n*=11)		Prevalence BKPyV-DNAemia patient (*n*=11)	
	MetaPhlAn4	Kaiju	MetaPhlAn4	Kaiju
*Citrobacter freundii*	36	100	0	100
*Escherichia coli*	27	100	9	100
*Finegoldia magna*	45	100	9	100
*Gardnerella vaginalis*	27	91	9	91
*Prevotella bivia*	9	100	0	100
*Serratia marcescens*	91	100	82	100
*Streptococcus mitis*	9	100	18	100
*Ureaplasma urealyticum*	9	55	0	55

## Discussion

In this study, we characterized, for the first time, the urinary microbiome in kidney transplant recipients with significant BKPyV-DNAemia. Our analyses included two bioinformatic tools to profile microbial taxonomy that utilize DNA-to-Marker (MetaPhlAn4) [32] and DNA-to-Protein approaches (Kaiju) [31]. Prior metagenomic benchmarking studies have indicated that DNA-to-Marker methods, such as MetaPhlAn4 typically yield high precision but poor sensitivity compared to Kaiju, which has high sensitivity but is prone to false positives and reduced precision [28]. Encouragingly, using these different bioinformatic approaches generated broadly similar microbiome signatures, providing greater confidence in our findings. Our characterization of urinary microbiome profiles supports findings of other studies based on enhanced urinary culture methods and DNA sequencing, which also found dominance of *Firmicutes*, *Proteobacteria* and *Actinobacteria* [23, 39–41]. At the genus level, both MetaPhlAn4 and Kaiju analyses showed a high prevalence and relative abundance of bacterial genera including *Serratia*, *Corynebacterium*, *Acinetobacter, Streptococcus*, *Citrobacter*, *Escherichia*, *Prevotella* and *Lactobacillus*. The similarities of these findings to other published studies further lend support to the urinary environment hosting a microbial community, although at low cell abundance. We found evidence of increased microbial taxonomic alpha diversity in the control group compared to patients with BKPyV reactivation, with significant differences observed from the MetaPhlAn4 data and non-significant but similar trends from the Kaiju dataset. Comparable patterns have been observed in other microbiome studies of viral infection and may be clinically relevant. For example, a diverse gut microbiota was shown to drive interferon-mediated antiviral responses and the secretion of immunoglobulin A in mice [20, 42–44], whilst specific cell wall components of *Lactobacillus brevis* may inhibit the replication of herpes simplex virus-2 [45]. Exploring whether greater urinary microbial diversity, or the presence of certain key taxa can drive enhanced local host antiviral responses or microbe-mediated protection against BKPyV-DNAemia is therefore an avenue for further investigation. Whether such responses would be effective under conditions of pharmacologically induced immunosuppression in transplant patients is an additional consideration.

Significant differences in taxonomic beta diversity were observed in control and BKPyV-DNAemia groups for the Kaiju dataset only. Since only the relative abundance of viral taxa (specifically Polyomaviruses) significantly differed between groups, it is likely that overall beta diversity profiles were driven by differential abundance of viruses or subtle changes in microbial abundance below the thresholds of detection in differential abundance tests. This hypothesis is further supported by the loss of significance in beta diversity group differences when viral reads were removed from the Kaiju data, in line with the results obtained from MetaPhlAn4.

Screening taxonomic data against a list of 243 bacterial pathogens taken from CZID [38] identified eight pathogens (*Citrobacter freundii*, *Escherichia coli*, *Finegoldia magna*, *Gardnerella vaginalis*, *Prevotella bivia*, *Serratia marcescens*, *Streptococcus mitis* and *Ureaplasma urealyticum*) common to both MetaPhlAn4 and Kaiju. Pathogen prevalences were generally higher from the Kaiju data; however, this may well be due to a higher rate of false positives [46] and so should be interpreted cautiously. Of the bacterial pathogens identified, *E. coli* was also cultured from one patient in the control group of this study. These pathogens have been commonly identified from urinary microbiome and cultures of renal transplant patients in other studies [47–50] and are typically associated with UTIs [51], which are a leading cause of sepsis in renal transplant patients [52, 53].

Ultimately, our characterization of differences in the urinary microbiome of BKPyV-DNAemia patients versus non-DNAemia controls points to new directions in biomarker discovery and advances in clinical understanding. Our study was, however, limited by its small sample size (22 patients) and poor recruitment of female patients, for which there is evidence for differences in urinary microbiome compared to men [15]. A wide range of patient ages were also included in our cohort, which given evidence of age-related changes in the urinary microbiome [40], may have added further challenges in identifying microbial biomarkers of pathology. We are also unable to rule out the effects of other key variables, such as immunosuppression, rejection history and transplant type on the urinary microbiome within our patient cohort. Finally, the lack of blank control samples during DNA extraction and library preparation risks contaminating sequences going unnoticed. This is especially likely to be a challenge for low DNA biomass samples, such as urine, which may bias our results [54]. Overall, these limitations prevent us from drawing broadly generalizable conclusions regarding the urinary microbiome and BKPyV-DNAemia. Future higher-powered studies should, therefore, seek to overcome these methodological and sampling limitations to determine the importance of sex, age and other clinically relevant variables in shaping the urinary microbiome and its potential link with BKPyV-DNAemia. Metagenomics paired with collection of microbial cultures will further help to determine whether the taxa identified here are present in the urinary environment as viable cells rather than DNA only, but will require specialist culture conditions to capture the diversity of fastidious taxa [55, 56]. More robust analyses of the virome that go beyond our investigation of DNA viruses will also be key in understanding the potential importance of other viral taxa in reactivation of BKPyV. Finally, sampling across multiple timepoints will be crucial to assess whether the microbiome is functionally important in BKPyV-DNAemia, diagnostic, or incidental to this pathology.

## Supplementary material

10.1099/jmm.0.002196 Supplementary Material 1.

## Abbreviations

AKI: acute kidney injury
BKPyV: BK polyomavirus
CRS: cardio-renal syndrome
DGF: delayed graft function
DM: diabetes mellitus
ESRD: end stage renal disease
FSGS: focal segmental glomerulonephritis
HD: haemodialysis
HRS: hepato-renal syndrome
HTN: hypertension
IFTA: interstitial fibrosis and tubular atrophy
N/A: not applicable
PyVAN: polyomavirus-associated nephropathy
SLE: systemic lupus nephritis
UTI: urinary tract infection
