# Supplementary material for: Urinary microbiome in renal transplant patients with BK polyomavirus reactivation
Source: J Med Microbiol. 2026 Sep 7;75(9):002196. doi: 10.1099/jmm.0.002196 (PMC13549444; doi:10.1099/jmm.0.002196)
Supplement: Supplementary file 1 [file jmm-75-02196-s001.pdf]

Supplementary Figures

Fig S1. Stacked bar plot of microbiome (> 0.1% mean relative abundance) analysed by Kaiju with non-bacterial taxa removed.

Fig S2. Stacked bar plot of bacterial taxa (> 0.1% mean relative abundance) identified from MetaPhlAn4.

Fig S3. Frequency of microbial taxa (> 0.1% mean relative abundance) identified from Kaiju and MetaPhlAn4 across samples.

Fig S4. Observed species diversity of microbial taxa identified from Kaiju and bacteria from MetaPhlAn4 (> 0.1% mean relative abundance).

SI Fig 1.

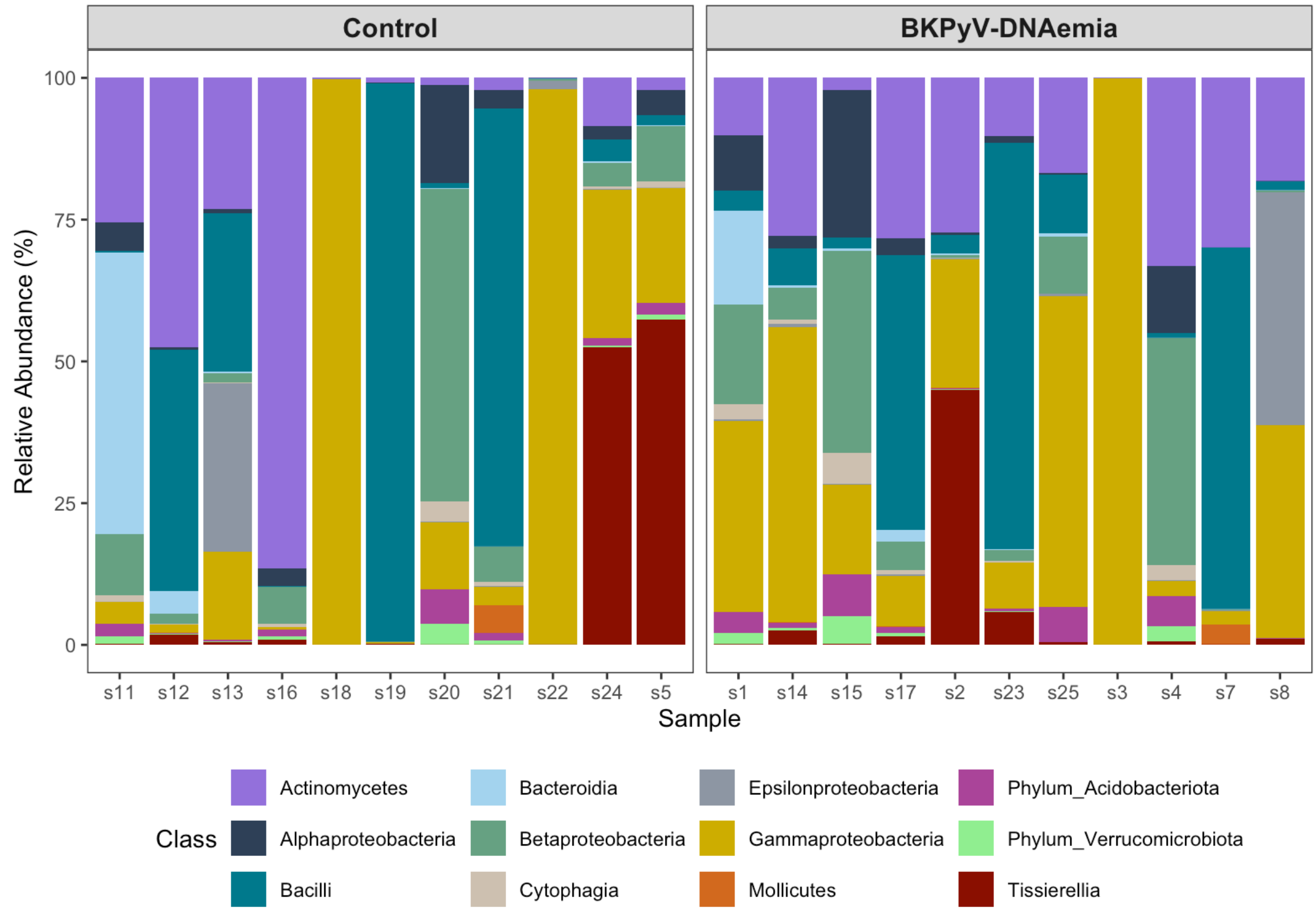

SI Fig 2.

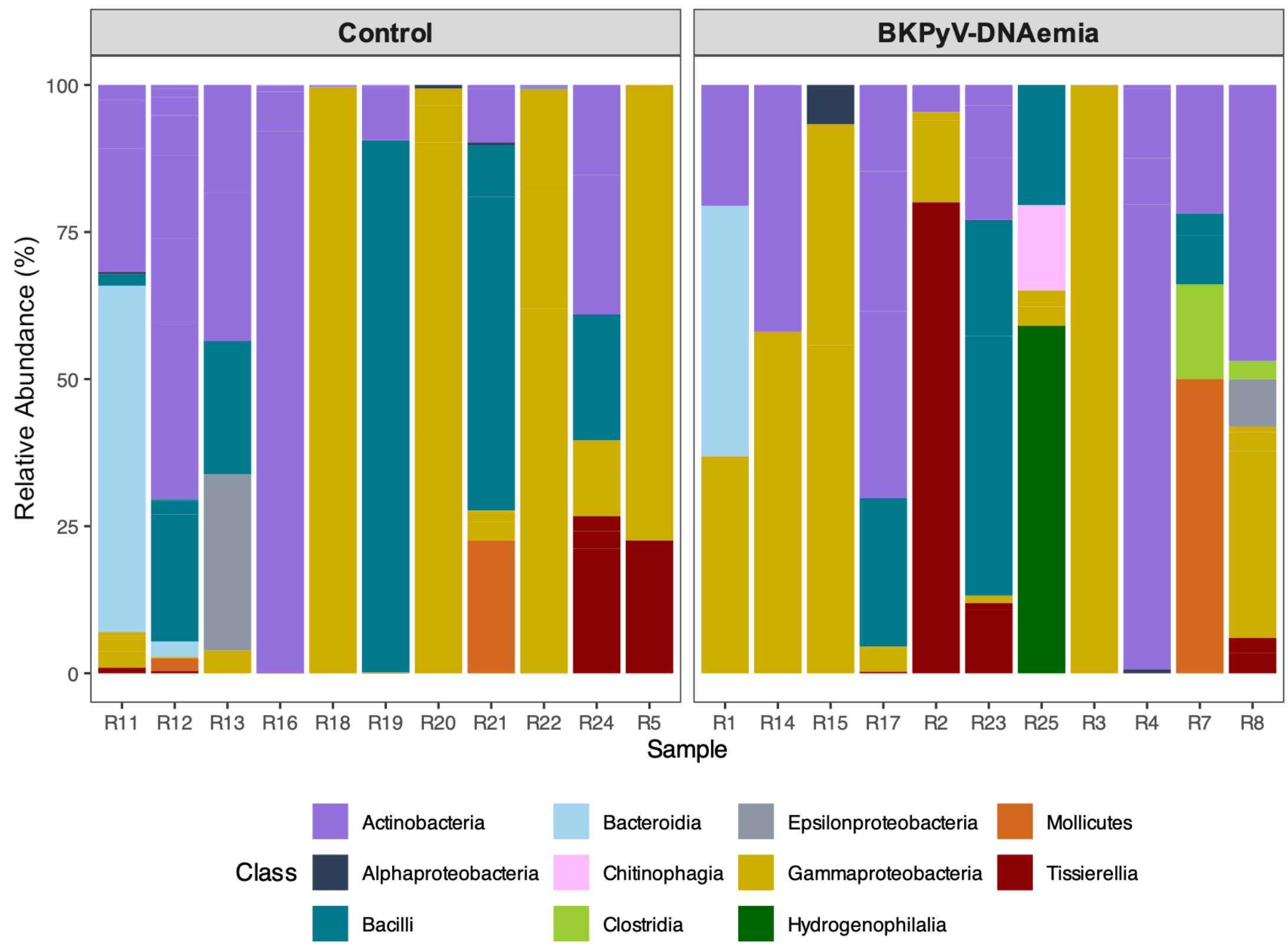

Fig S3.

Kaiju:

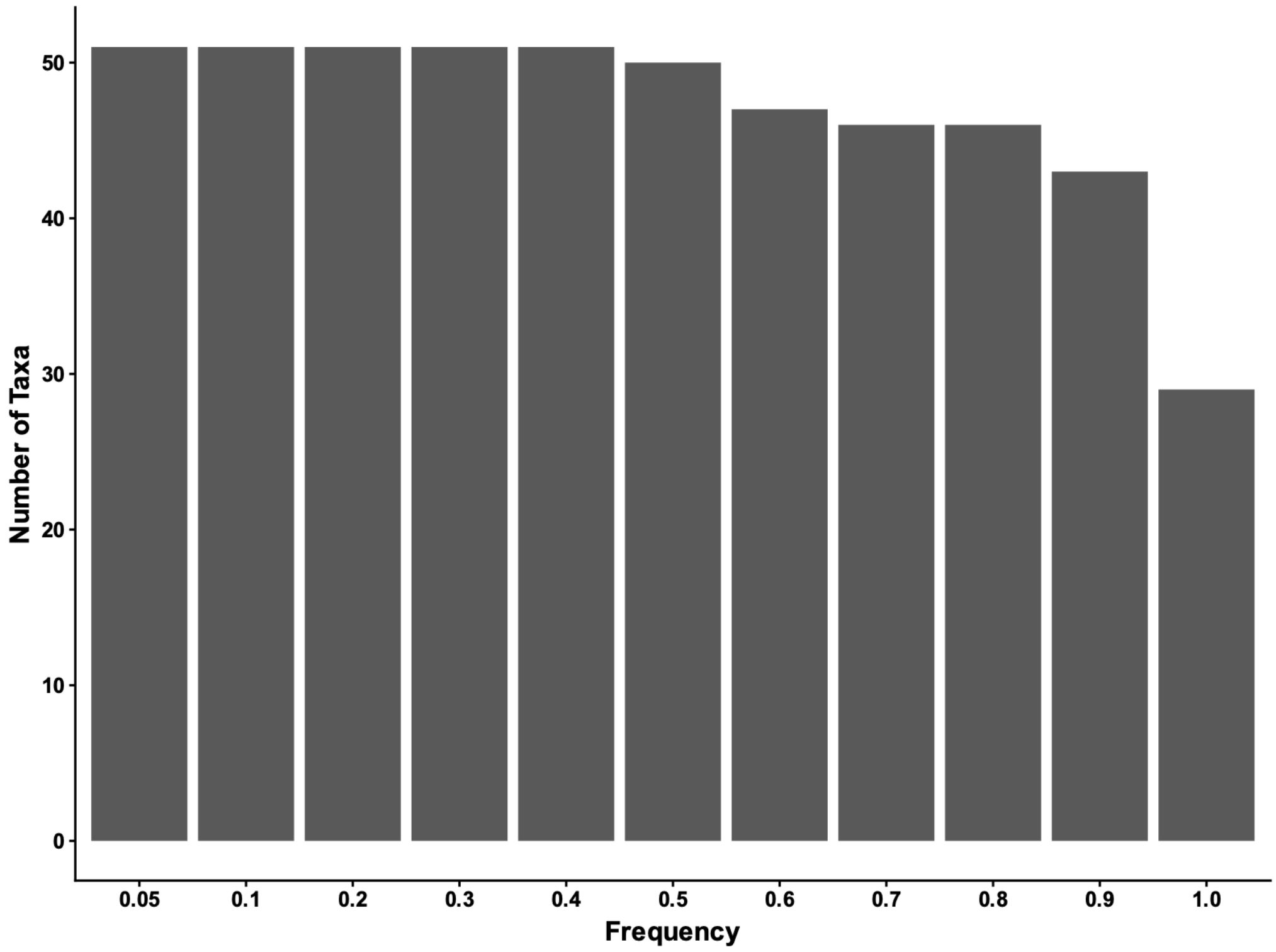

MetaPhlAn4:

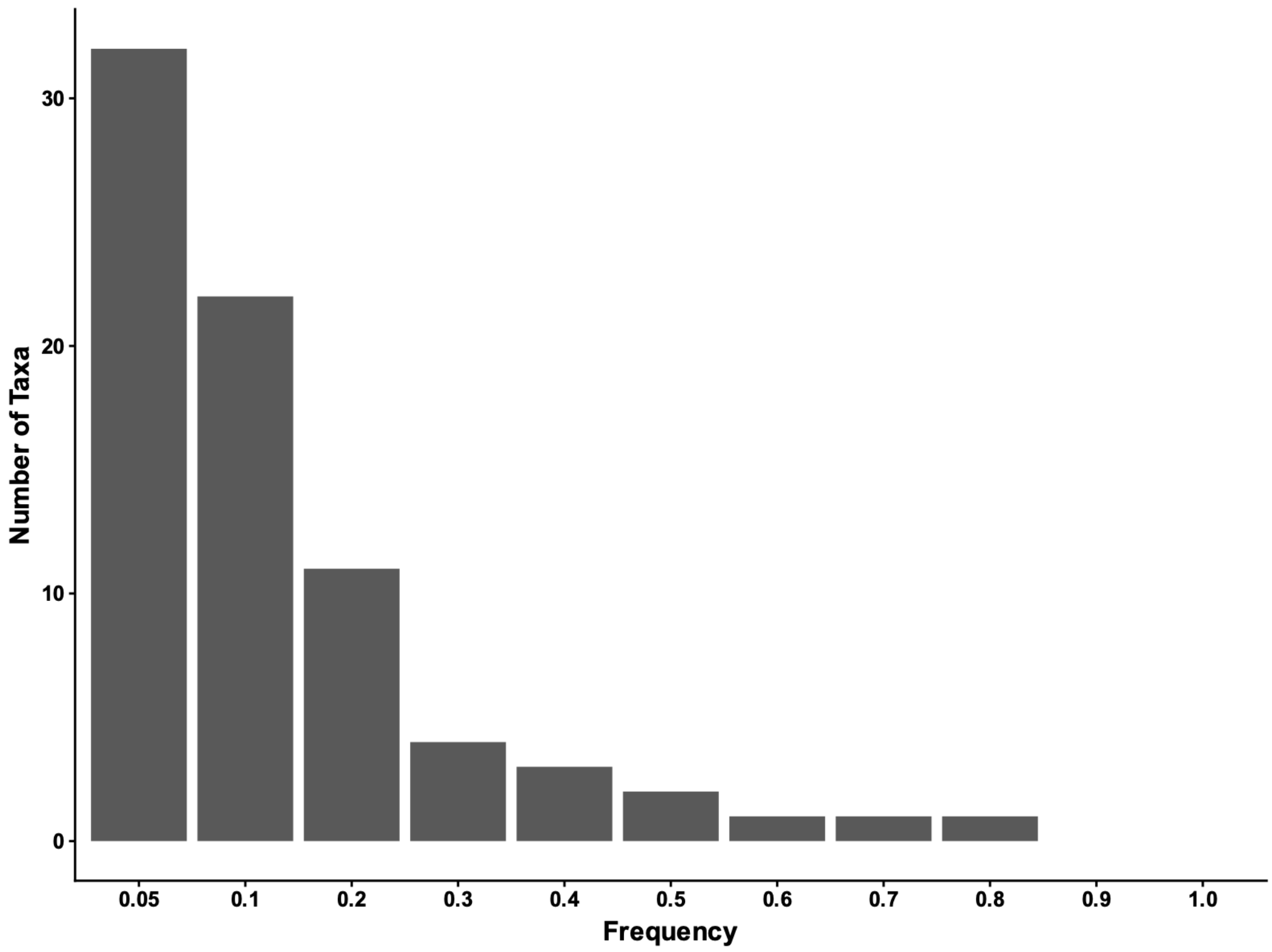

Fig S4.

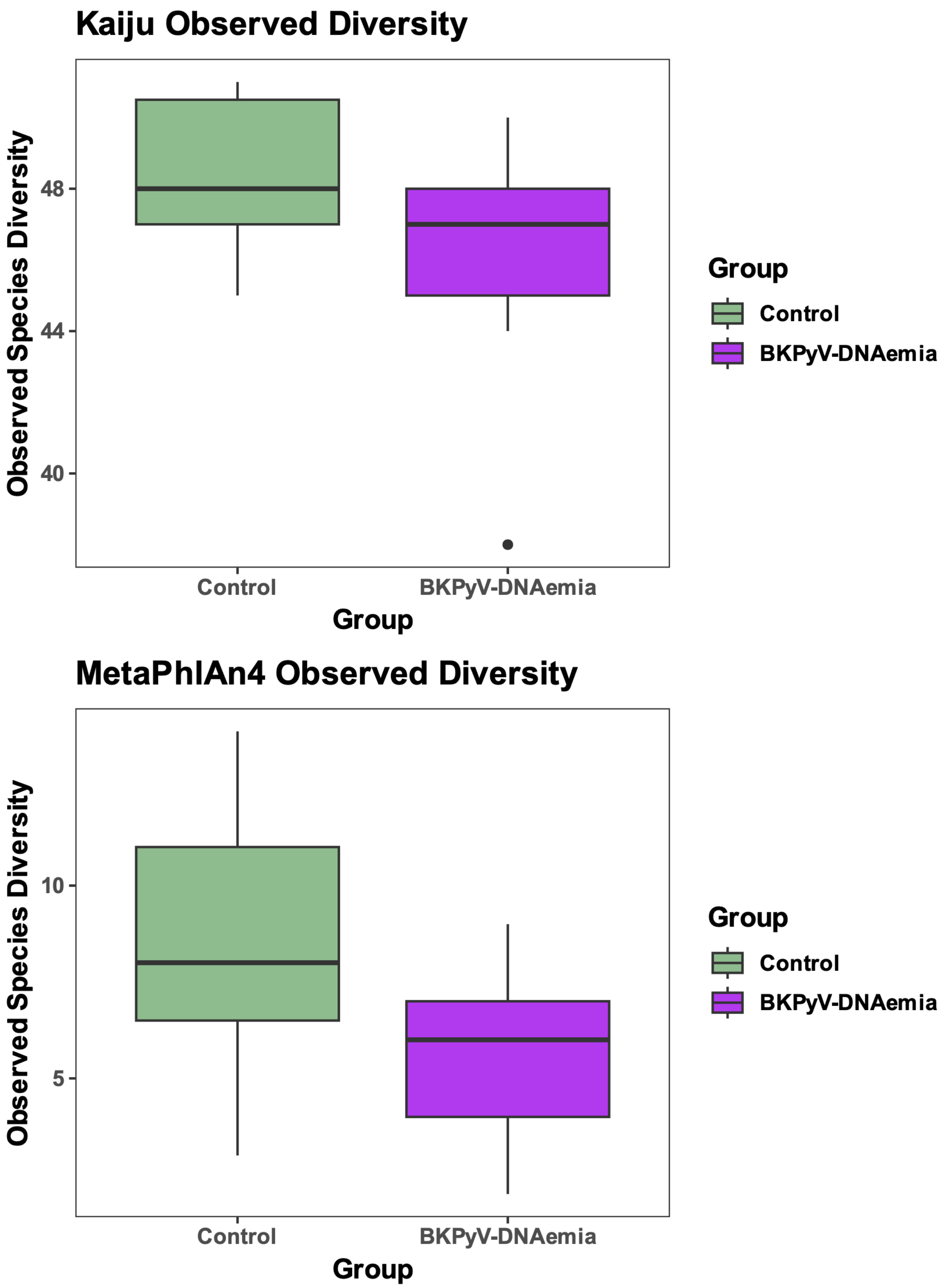
Kaiju Observed Diversity
Observed Species Diversity
48
44
40
Control
BKPyV-DNAemia
Group
Group
Control
BKPyV-DNAemia
MetaPhlAn4 Observed Diversity
Observed Species Diversity
10
5
Control
BKPyV-DNAemia
Group
Group
Control
BKPyV-DNAemia

Supplementary Tables

Table S1. ALDEx2 analysis of the Kaiju dataset

**Species**	**we.ep**	**we.eBH**	**wi.ep**	**wi.eBH**
Betapolyomavirus secuhominis	0.00116841	0.02884006	0.00013595	0.00630407
Family_Polyomaviridae	0.00226197	0.02884006	0.00043256	0.00747032
Genus_Betapolyomavirus	0.00161791	0.02884006	0.00055323	0.00761943
Betapolyomavirus hominis	0.00190463	0.02884006	0.00054472	0.00754647

Table S2. Biosample accessions for this study

**Accession**	**Sample**	**Name**	**SPUID Organism**	**Tax ID**	**BioProject**
SAMN48061795	R20	R20	urine metagenome	1346744	PRJNA1252894
SAMN48061796	R21	R21	urine metagenome	1346744	PRJNA1252894
SAMN48061797	R19	R19	urine metagenome	1346744	PRJNA1252894
SAMN48061798	R16	R16	urine metagenome	1346744	PRJNA1252894
SAMN48061799	R5	R5	urine metagenome	1346744	PRJNA1252894
SAMN48061800	R12	R12	urine metagenome	1346744	PRJNA1252894
SAMN48061801	R22	R22	urine metagenome	1346744	PRJNA1252894
SAMN48061802	R11	R11	urine metagenome	1346744	PRJNA1252894
SAMN48061803	R18	R18	urine metagenome	1346744	PRJNA1252894
SAMN48061804	R13	R13	urine metagenome	1346744	PRJNA1252894
SAMN48061805	R24	R24	urine metagenome	1346744	PRJNA1252894
SAMN48061806	R1	R1	urine metagenome	1346744	PRJNA1252894
SAMN48061807	R2	R2	urine metagenome	1346744	PRJNA1252894
SAMN48061808	R3	R3	urine metagenome	1346744	PRJNA1252894
SAMN48061809	R4	R4	urine metagenome	1346744	PRJNA1252894
SAMN48061810	R14	R14	urine metagenome	1346744	PRJNA1252894
SAMN48061811	R7	R7	urine metagenome	1346744	PRJNA1252894
SAMN48061812	R8	R8	urine metagenome	1346744	PRJNA1252894
SAMN48061813	R23	R23	urine metagenome	1346744	PRJNA1252894
SAMN48061814	R17	R17	urine metagenome	1346744	PRJNA1252894
SAMN48061815	R15	R15	urine metagenome	1346744	PRJNA1252894
SAMN48061816	R25	R25	urine metagenome	1346744	PRJNA1252894
